# Population attributable risks of oral cavity cancer to behavioral and medical risk factors in France: results of a large population-based case–control study, the ICARE study

**DOI:** 10.1186/s12885-015-1841-5

**Published:** 2015-10-31

**Authors:** Loredana Radoï, Gwenn Menvielle, Diane Cyr, Bénédicte Lapôtre-Ledoux, Isabelle Stücker, Danièle Luce, ICARE study group

**Affiliations:** 1 INSERM UMRS 1018, Environmental Epidemiology of Cancer, Centre for research in Epidemiology and Population Health, 16 Avenue Paul Vaillant Couturier, 94807 Villejuif Cedex, France; 2 Oral Medicine and Oral Surgery Department, Paris Descartes University, Montrouge, France; 3 INSERM, UMR_S 1136, Pierre Louis Institute of Epidemiology and Public Health, Paris, France; 4 Sorbonne Universités, UPMC Univ Paris 6, UMR_S 1136, Pierre Louis Institute of Epidemiology and Public Health, Paris, France; 5 INSERM UMS 011, Villejuif, France; 6 Versailles St-Quentin University, Versailles, France; 7 Registre du Cancer de la Somme, CHU Amiens, EA Inserm – DGS, EA 4666, Amiens, France; 8 Paris Sud University, Kremlin-Bicêtre, France; 9 INSERM U 1085, IRSET, Pointe-à-Pitre, French West Indies, Rennes, France; 10 University of Rennes 1, Rennes, France

**Keywords:** Oral cavity cancer, Population attributable risk, Tobacco, Alcohol, Tea, Body mass index

## Abstract

**Background:**

Population attributable risks (PARs) are useful tool to estimate the burden of risk factors in cancer incidence. Few studies estimated the PARs of oral cavity cancer to tobacco smoking alone, alcohol drinking alone and their joint consumption but none performed analysis stratified by subsite, gender or age. Among the suspected risk factors of oral cavity cancer, only PAR to a family history of head and neck cancer was reported in two studies. The purpose of this study was to estimate in France the PARs of oral cavity cancer to several recognized and suspected risk factors, overall and by subsite, gender and age.

**Methods:**

We analysed data from 689 oral cavity cancer cases and 3481 controls included in a population-based case–control study, the ICARE study. Unconditional logistic regression models were used to estimate odds ratios (ORs), PARs and 95 % confidence intervals (95 % CI).

**Results:**

The PARs were 0.3 % (95 % CI −3.9 %; +3.9 %) for alcohol alone, 12.7 % (6.9 %–18.0 %) for tobacco alone and 69.9 % (64.4 %–74.7 %) for their joint consumption. PAR to combined alcohol and tobacco consumption was 74 % (66.5 %–79.9 %) in men and 45.4 % (32.7 %–55.6 %) in women. Among suspected risk factors, body mass index 2 years before the interview <25 kg.m^−2^, never tea drinking and family history of head and neck cancer explained 35.3 % (25.7 %–43.6 %), 30.3 % (14.4 %–43.3 %) and 5.8 % (0.6 %–10.8 %) of cancer burden, respectively. About 93 % (88.3 %–95.6 %) of oral cavity cancers were explained by all risk factors, 94.3 % (88.4 %–97.2 %) in men and only 74.1 % (47.0 %–87.3 %) in women.

**Conclusion:**

Our study emphasizes the role of combined tobacco and alcohol consumption in the oral cavity cancer burden in France and gives an indication of the proportion of cases attributable to other risk factors. Most of oral cavity cancers are attributable to concurrent smoking and drinking and would be potentially preventable through smoking or drinking cessation. If the majority of cases are explained by recognized or suspected risk factors in men, a substantial number of cancers in women are probably due to still unexplored factors that remain to be clarified by future studies.

## Background

Oral cavity cancer [1] is an important cause of morbidity and mortality in France, with approximately 6000 new cases and 1500 deaths each year [2] and incidence rates among the highest in the world [3]. In France, the incidence of oral cavity cancer has been strongly decreasing among men and strongly increasing in women [4]. Changes in the prevalence of the main risk factors, i.e. a decrease of daily smoking in men and an increase in women, and a decrease in alcohol drinking in both genders, are likely to partially explain these trends [4]. Despite the high incidence of oral cavity cancer in France, the role of tobacco smoking and alcohol drinking has been rarely studied [5–8], while other potential risk factors (e.g. family history of head and neck cancer (HNC), body mass index (BMI), personal medical history, tea consumption) were examined only in the present case–control study [9–11].

Population attributable risks (PARs) are useful tool to estimate the burden of risk factors in cancer incidence. Although several epidemiological studies examined the joint effect of tobacco and alcohol consumption and found evidence of an interaction on an additive [12] or multiplicative [13–18] scale, only few of them estimated the proportion of HNC that can be attributable to tobacco consumption alone, alcohol consumption alone, and their combined consumption [13, 15, 17]. However, the understanding of the independent and joint effects of tobacco and alcohol could have important implications for prevention. A pooled analysis within the International Head and Neck Cancer Epidemiology (INHANCE) Consortium [15] and a European case–control study [13] reported similar PARs of oral cavity cancer to tobacco and/or alcohol consumption (PAR = 63.7 % and 61.3 %, respectively), but lower than that reported by one case–control study conducted in Latin America (PAR = 83.7 % for oral cavity and oropharynx) [16]. PARs of HNC to tobacco and alcohol differed by gender (greater in men that in women), and by age (greater in older subjects than in younger subjects) in three studies [13, 15, 19].

The few available studies on HNC related to alcohol and tobacco consumption which provided separate PARs of oral cavity cancer did not include French data, and none performed analysis on oral cavity cancers stratified by gender, age or anatomic location. However, concerning the carcinogenic effect of tobacco smoking and alcohol drinking, differences between oral subsites have been observed in some epidemiological studies [5, 20–25]. Moreover, among the other potential risk factors of HNC, only the PAR of HNC to a family history of HNC has been estimated. It was low, in the order of 2 % [26], although higher (23.2 %) in young adults with family history of early-onset cancer [19]. Estimates for PARs to other suspected risk factors were never provided.

In a previous analysis [5], we reported PARs for tobacco smoking and/or alcohol drinking for oral cavity subsites, but we did not assess the PAR due to the interaction between these factors, nor did we estimate PARs by gender and age. Also, it would be of interest to examine PARs of oral cavity cancer for other suspected risk factors, never provided previously.

Using data from the ICARE study, we conducted the present analysis to (i) estimate in France the proportion of oral cavity cancers attributable to the effect of tobacco smoking alone, alcohol drinking alone and to their joint effect; (ii) assess the PARs due to several potential risk factors previously observed in this study population (family history of HNC, personal history of oral candidiasis, low BMI and low tea consumption); (iii) examine differences in PARs, if any, by subsite, gender and age.

## Methods

### Study population

The study design, study population and data collection methods of the ICARE (Investigation of occupational and environmental CAuses of REspiratory cancers) study have been described in details elsewhere [27]. Briefly, the ICARE study is a multi-centre population-based case–control study on lung and HNC, carried out from 2002 to 2007 in 10 French administrative areas, covered by a general cancer registry.

The present analysis included only cases with oral cavity cancer (International Classification of Diseases 10^th^ revision [ICD-10] codes C01-C06 [1]) and all controls.

Cases eligibility criteria included histological confirmed primary malignant tumours of the oral cavity who were aged 75 or less at interview. Clinical and anatomopathological reports were reviewed to determine the topography and histological type of the tumours according to the International Classification of Diseases for Oncology [28]. Among the 1316 cases identified as eligible, 968 cases could be contacted, of which 792 (81.8 %) agreed to participate. Analyses were restricted to squamous cell carcinomas (772 cases). Controls were selected from the general population by random digit dialling, and were frequency-matched to the cases by age, gender and area of residence. Of the 4673 eligible controls, 4411 could be contacted, and 3555 (80.6 %) agreed to participate. All subjects gave their written informed consent for the participation in the study. The ICARE study was approved by the Institutional Review Board of the French National Institute of Health and Medical Research (IRB-Inserm, n° 01–036), and by the French Data Protection Authority (CNIL, n° 90120).

### Data collection

Subjects were interviewed face-to-face with standardized questionnaires by trained interviewers. The questionnaire included information about socio-demographic characteristics, medical and family history, detailed tobacco, alcohol and tea consumption (quantity, duration, type of product, age at starting, time since cessation), and anthropometric measurements (height, weight at interview, two years before and at age 30). Ever smokers were defined as subjects who had smoked at least 100 cigarettes in their lifetime, or who had smoked at least one pipe, cigar or cigarillo per week for at least one year. Ever alcohol and tea drinkers were defined as subjects who had consumed at least one drink per month for at least one year. To ascertain the medical history, study participants were asked if, throughout their lives, they had ever had one or more diseases among those mentioned in a list, including oral candidiasis. To ascertain the family history of cancer, subjects were asked if their biological mother and father and their full brothers or sisters had ever had a cancer. If the answer was “yes”, the subjects were asked to specify the type of cancer.

A summary version of the questionnaire was used when the subject was too sick to answer the complete questionnaire (83 cases (10.8 %) and 74 controls (2.1 %)). This questionnaire did not include information about anthropometric measurements, family and medical history, and tea consumption. Therefore, these subjects were excluded from the present analysis that finally included 689 cases and 3481 controls.

### Statistical analysis

Unconditional logistic regression models were used to estimate ORs, PARs and their 95 % CIs. The models included the variables age (< 45, 45–60 > 60 years), gender, area of residence (10 administrative areas), education level (primary or less, vocational secondary, general secondary and university), alcohol and tobacco consumption (never use of tobacco and alcohol as reference, tobacco but never alcohol use (tobacco alone), alcohol but never tobacco use (alcohol alone) and tobacco and alcohol use (joint effect)), BMI two years before the interview (< 25 kg.m^−2^/≥ 25 kg.m^−2^), family history of HNC in first degree relatives (yes/no), personal history of oral candidiasis (yes/no) and tea drinking (never/ever). The choice of adjusting for these variables was justified by our previous results that showed lower risks of oral cavity cancer in overweight/obese subjects compared to normal/underweight subjects [9] and in ever tea drinkers compared to never drinkers [11], and higher risks in subject with history of oral candidiasis or family history of HNC compared with subjects without history [10].

Logistic regression models were also used to determine the multiplicative interaction parameter Ψ and the 95 % CI by including the dummy variables ever tobacco consumption, ever alcohol consumption and their product term. An interaction parameter Ψ greater than 1 indicated an interaction between tobacco and alcohol consumption greater than one a multiplicative scale.

PARs and their 95 % CIs were calculated using the ‘aflogit’ procedure in STATA [29], which estimates the adjusted measures of population attributable fraction from a logistic regression model adapted to case–control studies, on the basis of the method of Greenland and Drescher [30].

Stratified analyses were conducted by gender and age (< 45, 45–60, > 60 years). Polytomous regression modes were used to estimate ORs, PARs and 95 % CI by subsite (base of the tongue, mobile tongue, gums, floor of the mouth, hard/soft palate, and other parts of the oral cavity).

All statistical tests were two-sided. Analyses were performed using the Stata Statistical Software release 10.0 (StataCorp 2007, College Station, Texas, USA).

## Results

Men represented more than two thirds of both cases and controls (78.2 % and 80.6 %, respectively). Cases were younger and had lower education level than controls (mean of age 56.8 and 58.5, respectively; university degree 12.3 % and 25.9 %, respectively). The most frequent tumour location was the floor of the mouth (27.7 %), followed by the mobile tongue (23.2 %), the base of the tongue (18.8 %), other parts of the mouth (11.5 %) and soft palate (10.8 %). Gums and hard palate represented only 5.7 % and respectively 2.3 % of cancer locations.

### PAR to tobacco and alcohol consumption (Table 1)

**Table 1:** Odds ratios (OR), population attributable risks (PAR) and confidence intervals (95 % CI) for oral cavity cancer associated with tobacco smoking, alcohol drinking and their joint effect, overall and by subsite, gender and age. ICARE study

	Cases	Controls	OR (95 % CI)^a^	PAR (95 % CI)^a^
	(*N* = 689)	(*N* = 3481)		
Oral cavity overall				
None consumption	37	926	reference	
Alcohol alone	14	297	1.1 (0.4–2.6)	0.3 % (−3.9–3.9)
Tobacco alone	135	1189	3.2 (1.9–5.3)	12.7 % (6.9–18.0)
Tobacco and alcohol	486	1040	17.3 (10.6–28.3)	69.9 % (64.4–74.7)
Total (Alcohol and/or tobacco)			Ψ = 5.2 (1.9–13.8)	82.9 % (73.8–88.5)
By subsite				
Base of tongue				
None consumption	5	926	reference	
Alcohol alone	5	297	0.8 (0.1–7.3)	-**
Tobacco alone	28	1189	3.3 (1.1–9.6)	14.5 % (0.6–26.5)
Tobacco and alcohol	90	1040	13.1 (4.6–37.3)	65.4 % (49.1–76.5)
Total (Alcohol and/or tobacco)			Ψ = 4.8 (0.5–46.1)	79.6 % (50.6–91.6)
Mobile tongue				
None consumption	16	926	reference	
Alcohol alone	3	297	0.6 (0.1–4.7)	-**
Tobacco alone	40	1189	3.1 (1.3–7.3)	16.5 % (3.5–27.7)
Tobacco and alcohol	100	1040	12.0 (5.1–28.5)	60.1 % (46.1–70.5)
Total (Alcohol and/or tobacco)			Ψ = 6.9 (0.8–60.5)	75.7 % (51.3–87.9)
Gum				
None consumption	5	926	reference	
Alcohol alone	2	297	0.8 (0.1–8.0)	-**
Tobacco alone	12	1189	1.5 (0.4–5.4)	11.1 % (−30.1–39.2)
Tobacco and alcohol	18	1040	2.6 (0.7–9.6)	26.2 % (−13.9–52.2)
Total (Alcohol and/or tobacco)			Ψ = 2.0 (0.2–23.4)	36.4 % (−62.3–75.0)
Floor of the mouth				
None consumption	2	926	reference	
Alcohol alone	2	297	2.4 (0.2–27.7)	0.5 % (−5.8–6.4)
Tobacco alone	33	1189	11.1 (2.5–48.7)	15.5 % (6.7–23.6)
Tobacco and alcohol	146	1040	88.1 (20.3–381.8)	79.6 % (70.8–85.7)
Total (Alcohol and/or tobacco)			Ψ = 3.3 (0.3–38.3)	95.6 % (82.3–98.9)
Soft palate				
None consumption	4	926	reference	
Alcohol alone	0	297	not estimated	
Tobacco alone	11	1189	2.0 (0.5–8.2)	7.2 % (−9.4–21.3)
Tobacco and alcohol	57	1040	17.5 (4.7–65.3)	75.1 % (55.1–86.1)
Total (Alcohol and/or tobacco)			Ψ not estimated	82.3 % (44.9–94.3)
Other parts of the mouth				
None consumption	5	926	reference	
Alcohol alone	2	297	4.4 (0.6–34.1)	3.4 % (−6.6–12.5)
Tobacco alone	10	1189	2.2 (0.4–12.8)	4.9 % (−8.5–16.6)
Tobacco and alcohol	61	1040	27.7 (5.7–135.1)	79.3 % (59.9–89.2)
Total (Alcohol and/or tobacco)			Ψ = 2.8 (0.3–26.5)	87.6 % (50.1–96.9)
By gender				
Male				
None consumption	11	482	reference	
Alcohol alone	9	247	0.7 (0.2–2.3)	–**
Tobacco alone	94	967	2.7 (1.3–5.4)	9.8 % (3.0–16.1)
Tobacco and alcohol	431	1008	13.2 (6.8–25.5)	74.0 % (66.5–79.9)
Total (Alcohol and/or tobacco)			Ψ = 7.2 (2.1–24.8)	83.3 % (68.8–91.1)
Female				
None consumption	26	444	reference	
Alcohol alone	5	50	1.4 (0.3–7.1)	0.9 % (−10.6–11.1)
Tobacco alone	41	222	3.6 (1.7–7.8)	22.4 % (6.8–35.5)
Tobacco and alcohol	55	32	41.9 (17.8–98.7)	45.4 % (32.7–55.6)
Total (Alcohol and/or tobacco)			Ψ = 8.0 (1.4–46.5)	68.7 % (49.4–80.6)
By age				
< 45 years				
None consumption	5	124	reference	
Alcohol alone	0	24	not estimated	
Tobacco alone	10	207	1.6 (0.3–8.8)	8.0 % (−29.8–34.8)
Tobacco and alcohol	33	62	31.5 (4.4–123.1)	67.7 % (41.5–82.1)
Total (Alcohol and/or tobacco)			Ψ not estimated	75.7 % (20.6–93.9)
45–60 years				
None consumption	15	300	reference	
Alcohol alone	3	97	0.4 (0.1–3.3)	-**
Tobacco alone	66	513	2.9 (1.4–6.0)	10.9 % (3.5–17.8)
Tobacco and alcohol	297	399	22.1 (10.9–44.7)	75.2 % (68.3–80.5)
Total (Alcohol and/or tobacco)			Ψ = 18.4 (2.2–152.3)	85.5 % (73.4–92.1)
> 60 years				
None consumption	17	502	reference	
Alcohol alone	11	176	2.4 (0.8–6.8)	2.5 % (−3.8–8.4)
Tobacco alone	59	469	5.1 (2.3–11.0)	16.8 % (7.9–24.7)
Tobacco and alcohol	156	579	16.4 (7.7–35.1)	62.8 % (52.7–70.8)
Total (Alcohol and/or tobacco)			Ψ = 1.4 (0.4–4.3)	82.1 % (67.4–90.2)

**Negative population attributable risk (PAR) (OR < 1 and not significant)

ORs and PARs for hard palate were not estimated because of lack of cases in the reference category (0 never drinker never smoker)

^a^Logistic model adjusted for age, gender, area of residence, education level, tobacco and alcohol consumption, BMI two years before the interview, family history of head and neck cancer, history of candidiasis and tea consumption

Ψ = alcohol – tobacco interaction term

ORs, 95 % CIs and PARs of oral cavity cancer to alcohol drinking alone, tobacco smoking alone and their joint consumption, and the multiplicative interaction parameter are presented in Table 1. Alcohol drinking alone was not associated with the risk of oral cavity cancer, while exclusive tobacco smoking and joint consumption of alcohol and tobacco increased the risk. The joint effect was greater than multiplicative (interaction parameter Ψ > 1). PARs were 0.3 % (95 % CI −3.9-3.9) for alcohol consumption alone, 12.7 % (6.9–18.0) for tobacco consumption alone, 69.9 % (64.4–74.7) for their joint consumption, and 82.9 % (73.8–88.5) for total consumption (alcohol and/or tobacco).

Differences between subsites were observed: the greatest PARs to tobacco consumption alone, joint and total consumption of alcohol and tobacco were observed for floor of the mouth cancer (15.5 % (6.7–23.6) for tobacco consumption alone, 79.6 % (70.8–85.7) for alcohol and tobacco consumption, and 95.6 % (82.3–98.9) for alcohol and/or tobacco consumption). The lowest PARs to tobacco consumption alone, joint and total consumption of alcohol and tobacco were found for gum cancer (11.1 % (−30.1–39.2) for tobacco consumption alone, 26.2 % (−13.9–52.2) for joint consumption of alcohol and tobacco, and 36.4 % (−62.3–75.0) for alcohol and/or tobacco consumption). For the base of the tongue and soft palate, oral subsites generally grouped with the oropharynx, the PARs were similar to that observed for mobile tongue and other parts of the mouth; for example, the total PAR was 79.6 % (50.6–91.6) for base of the tongue, 75.7 % (51.3–87.9) for mobile tongue, 82.3 % (44.9–94.3) for soft palate and 87.6 % (50.1–96.9) for other parts of the mouth. With respect to the exclusive alcohol drinking, the PARs were low and some negative values were found when the ORs were below 1 for base of the tongue, mobile tongue and gum cancers.

The PARs were lower in women than in men for both the joint and total consumption of alcohol and tobacco (in women: 45.4 %, 32.7–55.6, and 68.7 %, 49.4–80.6, respectively; in men: 74.0 %, 66.5–79.9, and 83.3 %, 68.8–91.1, respectively). Concerning the PARs stratified by age, it was difficult to conclude to any difference between subjects younger than 45 compared to subjects aged 45–60 or older because CIs are large and overlapped.

### PAR to other risk factors

**Table 2:** Odds ratios (OR), population attributable risks (PAR) and confidence intervals (95 % CI) for oral cavity cancer associated with body mass index, family history of head and neck cancer, history of oral candidiasis, and tea consumption, overall and by subsite, gender and age. ICARE study

		Cases	Controls	OR (95 % CI)^a^	PAR (95 % CI)^a^
		*N* = 689	*N* = 3481		
		E+/E-	E+/E-		
Body mass index 2 years before the interview <25 kg.m^−2^ vs. ≥25 kg.m^−2^					
Oral cavity overall		408/253	1405/1944	2.4 (1.8–3.0)	35.3 % (25.7–43.6)
By subsite	Base of tongue	67/56	1405/1944	2.9 (1.8–5.0)	42.4 % (20.5–58.2)
	Mobile tongue	91/64	1405/1944	1.9 (1.2–2.9)	25.8 % (5.1–42.1)
	Gums	23/13	1405/1944	4.2 (1.5–12.2)	55.1 % (4.7–78.9)
	Floor of mouth	110/71	1405/1944	2.4 (1.6–3.6)	35.3 % (17.9–49.1)
	Soft palate	49/22	1405/1944	2.6 (1.4–4.8)	37.6 % (9.6–56.9)
	Other parts of the mouth	56/22	1405/1944	2.7 (1.4–5.1)	39.1 % (10.1–58.8)
By gender	Male	332/203	1022/1600	2.8 (2.1–3.6)	39.9 % (30.0–48.3)
	Female	76/50	383/344	1.1 (0.6–2.0)	4.2 % (−38.4–33.7)
By age	<45	33/12	222/177	3.1 (1.0–9.1)	47.3 % (2.1–72.8)
	45–60	272/144	601/797	2.6 (1.8–3.6)	40.4 % (27.3–51.2)
	>60	103/97	582/970	2.1 (1.4–3.1)	26.7 % (11.3–39.5)
Family history of head and neck cancer (yes vs. no)					
Oral cavity overall		68/462	157/2784	1.9 (1.3–2.8)	5.8 % (0.6–10.8)
By subsite	Base of tongue	17/73	157/2784	2.6 (1.3–5.3)	11.2 % (−0.9–21.9)
	Mobile tongue	17/106	157/2784	2.5 (1.3–4.8)	8.6 % (−0.9–17.3)
	Gums	1/24	157/2784	0.9 (0.1–7.5)	- **
	Floor of mouth	13/138	157/2784	1.6 (0.8–3.3)	3.4 % (−5.0–11.1)
	Soft palate	9/50	157/2784	2.4 (0.9–6.1)	7.0 % (−6.1–18.6)
	Other parts of the mouth	5/58	157/2784	1.1 (0.3–3.3)	0.6 % (−12.5–12.2)
By gender	Male	54/375	120/2184	2.1 (1.4–3.3)	6.8 % (1.0–12.2)
	Female	10/87	37/600	1.3 (0.4–3.9)	2.1 % (−12.9–15.1)
By age	<45	3/35	16/353	4.9 (0.9–27.2)	8.5 % (−14.1–26.7)
	45–60	41/293	67/1189	2.0 (1.1–3.6)	6.7 % (−0.4–13.3)
	>60	20/134	74/1242	1.6 (0.8–3.0)	4.0 % (−4.1–11.5)
Personal history of oral candidiasis (yes vs. no)					
Oral cavity overall		14/645	80/3224	4.2 (1.7–10.5)	1.9 % (−2.1–5.7)
By subsite	Base of tongue	3/120	80/3224	1.7 (0.2–17.3)	0.6 % (−7.1–7.7)
	Mobile tongue	5/145	80/3224	9.2 (2.9–29.1)	4.9 % (−1.8–11.3)
	Gums	0/36	80/3224	not estimated	
	Floor of mouth	3/180	80/3224	3.9 (0.8–20.2)	1.2 % (−5.2–7.3)
	Soft palate	1/71	80/3224	3.3 (0.3–35.9)	1.4 % (−7.8–9.9)
	Other parts of the mouth	1/77	80/3224	3.6 (0.4–32.9)	1.6 % (−7.3–9.7)
By gender	Male	10/526	41/2548	2.9 (0.8–10.1)	1.2 % (−3.2–5.3)
	Female	4/119	39/676	5.2 (1.1–24.8)	4.6 % (−6.9–14.8)
By age	<45	0/41	21/389	not estimated	
	45–60	10/403	27/1341	9.8 (2.7–35.9)	2.9 % (−2.2–7.8)
	>60	4/201	32/1494	1.6 (0.3–8.6)	0.6 % (−5.1–5.9)
Tea consumption (never vs. ever)					
Oral cavity overall		443/150	2025/1334	1.7 (1.3–2.2)	30.3 % (14.4–43.3)
By subsite	Base of tongue	80/37	2025/1334	1.0 (0.6–1.7)	0.1 % (−44.6–30.9)
	Mobile tongue	102/36	2025/1334	1.5 (0.9–2.5)	23.2 % (−13.2–47.9)
	Gums	20/10	2025/1334	1.9 (0.6–6.0)	36.1 % (−57.3–74.0)
	Floor of mouth	128/38	2025/1334	1.5 (0.9–2.3)	23.6 % (−7.1–45.4)
	Soft palate	48/13	2025/1334	4.3 (1.7–10.7)	67.5 % (29.3–85.0)
	Other parts of the mouth	55/13	2025/1334	4.2 (1.6–11.1)	67.6 % (24.1–86.2)
By gender	Male	377/99	1692/928	1.9 (1.4–2.6)	38.0 % (21.3–51.2)
	Female	66/51	333/406	1.3 (0.7–2.4)	13.9 % (−18.1–37.3)
By age	<45	231/172	28/14	2.3 (0.7–7.8)	45.7 % (−37.1–78.5)
	45–60	848/561	284/93	1.4 (0.9–2.1)	22.2 % (−3.22–41.2)
	>60	946/601	131/43	1.9 (1.3–3.1)	38.2 % (13.0–56.1)

**Negative population attributable risk (OR < 1 and not significant)

^a^Logistic model adjusted for age, gender, area of residence, education level, tobacco and alcohol consumption, BMI 2 years before the interview, family history of head and neck cancer, history of candidiasis and tea consumption

E+ exposed subject/E- non-exposed subject to a risk factor

ORs, PARs and 95 % CI for oral cavity cancer associated with other risk factors than alcohol and tobacco are presented in Table 2. Around 35 % (25.7–43.6) of oral cavity cancer cases were attributable to a BMI < 25.0 kg.m^−2^. Since the confidence intervals overlapped, it is not possible to conclude to significant differences between subsites. Among men, the corresponding PAR was 39.9 % (30.0–48.3). No association was observed in women between BMI and oral cavity cancer (OR = 1.1 (0.6–2.0)), leading to a small PAR (4.2 %, −38.4–33.7). No significant differences were observed in PAR stratified by age.

Only few oral cavity cancers were attributable to having a family history of HNC in first degree relatives (5.8 %, 0.6–10.8). No noticeable differences were observed in PAR by subsite or by age. The PARs were 6.8 % in men and 2.1 % in women with overlapping CIs.

Very few oral cancer cases were attributable to a personal history of oral candidiasis (1.9 %, −2.1–5.7 %). We did not find any significant difference in PARs stratified by subsite, gender or age.

The PAR associated with never drinking tea was 30.3 % (14.4–43.3). The highest ORs and PARs were observed for soft palate and other parts of the mouth but confidence intervals were large and overlapped and it was difficult to conclude to any difference. The PAR was 38.0 % in men and only 13.9 % in women, confidence intervals overlapping. Analysis stratified by age did not show significant differences in PAR.

### PAR to all risk factors combined

**Table 3:** Population attributable risks (PAR) and their confidence intervals (95 % CI) for oral cavity cancer associated with all risk factors, overall, by subsite, gender and age. ICARE study

		PAR (95 % CI)^a^
Oral cavity overall		92.8 % (88.3–95.6)
By subsite	Base of tongue	89.9 % (71.6–96.4)
	Mobile tongue	88.1 % (72.4–94.9)
	Gums	78.5 % (15.2–94.6)
	Floor of mouth	98.0 % (91.4–99.5)
	Soft palate	96.8 % (86.3–99.3)
	Other parts of the mouth	97.5 % (86.9–99.5)
By gender	Male	94.3 % (88.4–97.2)
	Female	74.1 % (47.0–87.3)
By age	<45	94.3 % (59.4–99.2)
	45–60	93.7 % (87.3–96.9)
	>60	92.4 % (84.2–96.4)

^a^Logistic model adjusted for age, gender, area of residence, education level, tobacco and alcohol consumption, BMI 2 years before the interview, family history of head and neck cancer, history of candidiasis and tea consumption

The PAR to all factors combined was around 93 % (95 % CI 88.3–95.6) with the lowest value of 78.5 % (95 % CI 15.2–94.6) for the gum cancer and the highest value of 98.0 % (95 % CI 91.4–99.5) for the floor of the mouth cancer (Table 3). The PARs varied by gender, the studied risk factors explaining 94.3 % (95 % CI 88.4–97.2) of cases in men, and only 74.1 % (95 % CI 47.0–87.3) of them in women, mainly because of differences between the two genders in risks related to tobacco and alcohol consumption. No significant difference in PAR by age was found.

## Discussion

The ICARE study is one of the few studies investigating the PARs of oral cavity cancer to several recognized or suspected risk factors. The PARs should in principle be estimated for risk factors with a proven causal relationship with a cancer/the disease. Nevertheless, we also calculated PARs for several suspected risk factors. Some of these factors (family and medical history) are not modifiable or can hardly be subject to preventive measures. In addition, obviously, it is not possible to recommend a weight gain that was inversely associated with the risk oral cavity cancer, because of the negative consequences on many other diseases. However, the objective here was to assess the impact of each risk factor, prioritize them, and determine the proportion of oral cavity cancers that remains to be possibly explained by other unexplored factors.

Our results have shown that the proportion of risk attributable to tobacco smoking alone was greater than that attributable to alcohol drinking alone. Smoking was an independent risk factor for oral cavity cancer while drinking, in the absence of smoking, conferred little and no significant risk. These results are similar to those reported in other studies [12, 13, 15, 17, 18, 31, 32]. Consistently with the difference in risks associated with smoking or smoking and drinking by subsite found in our study [5] as elsewhere [20–25], we observed differences in the estimates of PARs across oral cavity subsites. Some negative estimates for the PAR to exclusive alcohol drinking were observed. This does not indicate a protective effect of alcohol since the corresponding ORs did not suggest statistically significant inverse associations. We also observed a greater than multiplicative interaction between tobacco and alcohol, consistent with previous studies [13–18].

The proportion of cases attributable to the joint effect of tobacco and alcohol was around 70 %, confirming that tobacco and alcohol together explain the majority of oral cavity cancer burden in France. This result is consistent with that reported by one case–control study conducted in Latin America [17], but higher than that observed in an international pooled analysis [15] and a European case–control study [13].

Differences in PARs by gender were observed, particularly with regards to the attributable risks to tobacco and alcohol. The PAR to their joint consumption was higher in men than in women (74.0 % and 45.4 %, respectively), consistent with previous studies [13, 15, 17]. This can be explained by the higher proportion of drinkers and/or smokers in men than in women; the prevalence of combined consumption was around 78 % in male cases and only 41 % in female cases. Nevertheless, we cannot rule out more underreporting of these expositions among women than among men, especially for alcohol drinking, which is less socially accepted among women.

Conversely to the available studies [13, 15, 19], we did not find a lower proportion of oral cavity cancers attributable to alcohol and tobacco consumption in subjects younger than 45, probably because of the small size of this category compared to the older age groups.

In our study, PAR of oral cavity cancer to family history of HNC was around 6 %, greater than that reported for HNC overall in the pooled analysis within INHANCE Consortium (around 2 %) [26]. This difference may be explained by the higher percentage of subjects exposed to a family history of HNC in our study (9.9 % in cases and 4.5 % in controls) than in the pooled analysis (3.6 % in cases and 1.8 % in controls).

Low versus high BMI and never versus ever consumption of tea appear to be responsible for a significant number of oral cavity cancers, especially in men. The possible role of body fat in the distribution of the lipophilic carcinogens derived from tobacco smoke [33] and the implication of tea polyphenols in the apoptosis and the inhibition of the growth of oral carcinoma cells [34, 35] might explain these findings.

Approximately 93 % of cases of oral cavity cancer overall, 94 % in men and only 74 % in women, are explained by all the studied factors. This leaves about 7 % of cases to be explained by other risk factors that we have not been able to take into account, of which 6 % in men and 26 % in women. This result emphasizes the role of other risk factors in women, such as human papillomavirus (HPV) infection, diet, hormonal factors or specific genetic factors of cancer susceptibility.

Strengths of our study are the multicenter design and the study size that allowed us to perform stratified analyses by anatomic subsite, gender and age.

Some limitations of our study exist. The subjects self-reported their own consumption of tobacco, alcohol and tea, anthropometric measurements, medical and family history. Thereby, recall bias could not be ruled out and it is possible that the cases had a higher motivation than the controls to recall tobacco and alcohol consumption as well as medical and family history, known as potential risk factors of cancer. Nevertheless, we do not think that underreporting of alcohol consumption may explain its negligible impact because alcohol drinking is socially accepted in France. In France, only 8.4 % of people reported never having drunk alcohol [36], a proportion close to that of never drinkers among our controls (8.6 %). We think that recall bias for BMI and tea drinking would be non-differential among cases and controls, since these factors are not generally known to be related to oral cavity cancer. If present, this bias would tend to attenuate the associations with cancer risk. In addition, many studies have shown that subjects in case–control studies are able to accurately self-report family history of common types of cancer among first-degree relatives [37, 38] and tea consumption [39].

Information about other suspected or known risk factors for oral cavity cancer such as HPV infection or diet was not collected in our study and residual confounding cannot be excluded. HPV infection is a recognized risk factor for base of the tongue and tonsils cancers, but less for the other HNC [40]. However, adjustment for HPV seemed to have only minor effects on the estimated ORs for alcohol and tobacco [41–43]. In addition, an International Agency for Cancer Research report on attributable causes of cancer in France estimated the PAR of oral cavity and oropharynx cancers to HPV infection low and similar in both genders (6.7 %) [44]. A diet rich in fruits and vegetables, generally associated with lower body size, have a protective effect against HNC [45–47]. Thus, confounding by diet is unlikely to explain the inverse association between BMI and oral cavity cancer that we found, although it could reduce the precision of the ORs. Also, we think that the lack of information on HPV and diet could not explain the differences in PARs that we observed.

## Conclusion

While we did not observe an independent effect of alcohol on oral cavity cancer risk in our study, concurrent smoking and drinking are responsible for the majority of oral cavity cancers, especially among men. In terms of public health, this study suggests that a reduction of combined tobacco and alcohol consumption could result in a significant decrease of oral cancer. Ninety-three percent of cases overall and 94 % in men are explained by known or suspected risk factors. However, a substantial proportion of oral cavity cancer among women (26 %) cannot be attributed to either established or suspected risk factors. Still unexplored factors conferring oral cancer risk among women remain to be clarified by future studies.

## Abbreviations

PAR: population attributable risk
OR: odds ratio
95 % CI: 95 % confidence interval
HNC: head and neck cancer
BMI: body mass index
HPV: human papillomavirus
INHANCE consortium: International Head and Neck Cancer Epidemiology Consortium
ICARE study: Investigation of occupational and environmental CAuses of REspiratory cancers study
ICD: International Classification of Diseases
